# Polyacrylonitrile Ultrafiltration Membrane for Separation of Used Engine Oil

**DOI:** 10.3390/polym16202910

**Published:** 2024-10-16

**Authors:** Alexandra Nebesskaya, Anastasia Kanateva, Roman Borisov, Alexey Yushkin, Vladimir Volkov, Alexey Volkov

**Affiliations:** Laboratory of Polymeric Membranes, A.V. Topchiev Institute of Petrochemical Synthesis Russian Academy of Sciences, 119991 Moscow, Russia; kanatieva@ips.ac.ru (A.K.); borisov@ips.ac.ru (R.B.); vvvolkov@ips.ac.ru (V.V.); avolkov@ips.ac.ru (A.V.)

**Keywords:** ultrafiltration, used engine oil, membrane, poly(acrylonitrile-co-methyl acrylate), separation

## Abstract

The separation of used engine oil (UEO) with an ultrafiltration (UF) membrane made of commercial copolymer of poly(acrylonitrile-co-methyl acrylate) (P(AN-co-MA)) has been investigated. The P(AN-co-MA) sample was characterized by using FTIR spectroscopy, ^13^C NMR spectroscopy, and XRD. The UF membrane with a mean pore size of 23 nm was fabricated by using of non-solvent-induced phase separation method—the casting solution of 13 wt.% P(AN-co-MA) in dimethylsulfoxide (DMSO) was precipitated in the water bath. Before the experiment, the used engine oil was diluted with toluene, and the resulting UEO solution in toluene (100 g/L) was filtered through the UF membrane in the dead-end filtration mode. Special attention was given to the evaluation of membrane fouling; for instance, the permeability of UEO solution was dropped from its initial value of 2.90 L/(m^2^·h·bar) and then leveled off at 0.75 L/(m^2^·h·bar). However, the membrane cleaning (washing with toluene) allowed a recovery of 79% of the initial pure toluene flux (flux recovery ratio), indicating quite attractive membrane resistance toward irreversible fouling with engine oil components. The analysis of the feed, retentate, and permeate by various analytical methods showed that the filtration through the UF membrane made of P(AN-co-MA) provided the removal of major contaminants of used engine oil including polymerization products and metals (rejection—96.3%).

## 1. Introduction

In recent years, there has been a significant increase in interest in developing technologies for the reuse of waste lube oils. The analysis of scientific publications from 1997–2017 on the topic of “waste engine oil” demonstrated a sharp increase in the number of publications starting in 2008 [1]. This research area remains relevant in recent years (see, for example, recent reviews [2,3,4,5,6,7,8,9,10,11,12]). In 2017, the world lubricant market was estimated at around 35.7 million metric tons [4]. According to other estimates, the total demand for lubricants in 2020 was 31 million metric tons, with global lubricant demand potentially reaching 37.4 million metric tons in 2023 [12]. It is important to note that the dynamics of lubricant oil demand vary across different regions of the world. From 2007 to 2017, demand for global lubricant oil consumption in the Asia-Pacific region increased from 35% to 43%, while in North America and Europe, demand decreased from 22% to 18% and from 23% to 19%, respectively [4]. Generally, lubricant oil comprises 70–90% base oil, and the remainder consists of additives [10].

During use, lubricating oils lose their antifriction properties, leading to the generation of significant waste. Used lubricating oils are considered hazardous waste and have a tremendous impact on human health and the environment. They contain heavy metals (e.g., Cr, Cd, As, and Pb) and harmful chemical compounds, such as benzene, chlorinated solvents, polychlorinated biphenyl, and polycyclic aromatic hydrocarbons [7].

Besides posing a serious environmental hazard, used lubricants are a valuable secondary raw material that should be collected and recycled. Over the past decades, numerous methods for recycling used oils have been developed. The most well-known traditional methods include (i) acid-clay and its modification, (ii) vacuum distillation and its upgrading, (iii) hydro-treating, and (iv) extraction-flocculation with an integrated adsorption process [10,12]. However, approximately only 35% of the collected oil is refined into base oil; the remaining 65% is burned as a coal replacement (10%), used as heavy fuel oil (45%), and other unknown products (10%) [13]. At the same time, common technologies for re-refining waste lube oils are relatively expensive due to high energy consumption and secondary pollution [1].

As an alternative approach, the use of membrane technologies, primarily micro- and ultrafiltration (UF), is being explored for used oils recycling [1,2,3]. Since the driving force of these processes is the pressure difference across the membrane, separation occurs continuously and under relatively mild conditions without phase transitions, allowing liquid systems to be separated with lower energy costs compared to traditional distillation. However, the high viscosity of engine oil is a limiting factor for the application of filtration processes for its regeneration. Reducing the viscosity of the initial mixture can be achieved by filtration at elevated temperatures or by preliminary dilution of the initial mixture with a solvent. Both of these approaches were initially successfully used in the membrane separation processes of crude oil: filtration at elevated temperatures of 80–190 °C [14] and filtration of oil diluted with naphtha [15].

For the filtration of used oil at elevated temperatures, ceramic membranes are most commonly used. For instance, in [16], the regeneration of used transformer and motor oils was studied on industrial installations using laboratory samples of ceramic membranes at operating pressures of 0.4–0.6 MPa and temperatures of 50–80 °C. During operation, the membrane pores were significantly fouled by components of the filtered oil, leading to increased selectivity (regeneration degree) and a significant decrease in permeability in the first 3–4 h of operation. The fouling issue was resolved by applying cyclic membrane regeneration with compressed air at a pressure of 0.6 MPa, restoring the target characteristics of the membranes to 85–90% after each regeneration cycle [16].

The treatment of used oil in cross-flow filtration mode using microfiltration ceramic membranes was studied in [17]. A module based on ceramic tubular membranes was provided by Tami Deutschland GmbH. The ash content in the burned permeate was reduced by at least 65% compared to the original oil. Direct and reverse washing of the membranes partially restored the enrichment factor to 80% of the initial value.

The unique combination of properties of ceramic membranes—thermal stability and mechanical strength—was successfully utilized by Rios and coworkers [18,19,20,21]. The authors proposed using supercritical CO_2_ as a diluent to reduce the viscosity of used oil and, as a result, increase the flux through the membrane. The regeneration of used oils and other high-viscosity liquids was studied using cross-flow ultrafiltration enhanced by the addition of supercritical CO_2_. It was shown that the higher the CO_2_ pressure, the lower the viscosity, and the lower the temperature, the greater the viscosity reduction effect. The experimental setup allowed for research at pressures and temperatures up to 200 bar and 150 °C, making the choice of ceramic membranes ideal for operating under such harsh conditions without membrane degradation. The achieved viscosity reduction allowed for a fourfold increase in effective permeate flux. At the same time, metal rejection rates always exceeded 95% [20].

In the study [22], a hybrid coagulation–ultrafiltration process for used engine oils (UEO) on tubular ceramic membranes was investigated. It was noted that coagulation, due to particle aggregation and organic substances, contributes to the formation of a more porous cake layer, reducing pore blockage and enhancing filtration efficiency. The filtration process was conducted in a membrane module consisting of a single tubular ceramic element with pore sizes of 0.1–0.01 µm. The transmembrane pressure was maintained at 0.1–0.5 MPa. The effectiveness of the hybrid process was confirmed by the composition and properties of the regenerated oil: metal content decreased by 2–5 times, asphaltene and resinous aging products were virtually absent, and both viscosity and the base number, indicating the removal of used viscous additives from the oil, were reduced.

The use of polymer membranes, particularly high-temperature polymer membranes, offers significant advantages over costly ceramic membranes. Overall, in recent decades, polymeric materials have been increasingly used in various sectors of the chemical industry, such as pharmaceuticals [23,24], electrochemistry [25,26,27], as well as in gas separation [28,29,30] and pervaporation [29,31], wastewater treatment [24,32], and the production of food and beverages [24]. Research on the development of polymer-based membranes for oil [33,34,35,36,37] and petroleum products [38,39,40,41,42,43,44] separation has also garnered significant attention. Accordingly, a number of flat-sheet and hollow fiber membranes based on traditional polymeric materials, specifically polyethersulphone (PES) [39], polyvinylidenefluoride (PVDF) [39,40], polyacrylonitrile (PAN) [39], polyimide (PI) [41], polypropylene (PP) [42,43], and polytetrafluoroethylene (PTFE) [44], were studied for used oil processing.

In [39], three types of hollow fiber membranes—PES with a pore size of 0.1 µm, PVDF with a pore size of 0.1 µm, and PAN with the molecular weight cut-off (MWCO) of 50 kDa—were used to separate waste lubricant oil. Filtration was performed at 40 °C and 0.1 MPa. As expected, the PAN ultrafiltration membrane exhibited a higher degree of purification than the PES and PVDF microfiltration membranes. The rejection of the PAN membrane was 99.6%. Analysis of the retentate composition revealed that it mainly retained aromatic compounds, oxidation, and degradation products, as well as metal particles, carbon dust, etc.

Comparable performance values were obtained in [43] when filtering waste engine oil using polypropylene (PP) hollow fiber ultrafiltration membranes with a pore size of 0.05 µm. The permeate fluxes obtained for the PP membrane at an operating pressure of 1 bar and 50 °C were comparable to the data for the PAN ultrafiltration membrane [39]. Quality analysis of the treated oil indicated that the PP hollow fiber membrane effectively removed contaminants from used engine oil.

There are also publications on the development of thermally stable polymer membranes for the regeneration of used oil at elevated temperatures. For example, in [44], reinforced hollow fiber composite membranes PTFE/GE (graphene) with high-temperature resistance were designed. First, the GE was attached to the glass fiber braided tube with a diameter of 2.5 mm (according to SEM data). Afterward, the biaxially stretched PTFE flat-sheet membranes were pressed on the surface of the reinforced GE membrane to prepare the PTFE/GE HFCMs. Filtration experiments used lubricants with added activated carbon particles to evaluate membrane separation characteristics. The separation characteristics of the membranes were studied at temperatures of 60, 90, 120, and 150 °C. Membrane permeability initially decreased significantly and then stabilized after approximately 12 h of operation, with rejection rates remaining stable above 99% for lubricants containing activated carbon.

In [40], composite flat-sheet ultrafiltration membranes made from PVDF with added ground large particle glass fibers were investigated. The PVDF and PVDF-glass fiber (PGF) composite flat membranes were prepared using the non-solvent induced phase separation (NIPS) method. Used engine oil filtration was conducted in a flow-through setup at 4 bar and 80 °C for 60 min, followed by regeneration of fouled membranes by washing with gasoline. Metal rejection from the composite membrane was approximately equal to that of the PVDF membrane. Furthermore, a comparison of the physicochemical properties of the used and filtered oil showed a high degree of regeneration for both membranes; however, the viscosity index and flash point were higher when using the PVDF-glass fiber composite membrane.

In 2016, New Logic Research Inc. published an economic assessment of the efficiency of the used oil regeneration process using the V-SEP (Vibratory Shear Enhanced Process) separation system based on polymer membranes, showing that this process is promising and profitable [45]. The plant uses a single high-temperature microfiltration module with an area of 100 m^2^, capable of processing over 20,000 gallons of used lubricating oils in just 1.75 days. The permeate quality produced at the plant is comparable to marine-grade diesel fuel or bunker fuel. However, the issue of the short lifespan of polymer membranes and the selection of regeneration methods remains unresolved.

The most outstanding result in the field of creating chemically and thermally resistant polymer membranes is polytriazole nanofiltration membranes [34]. Flat polytriazole membranes with 10-nanometer-thin selective layers were fabricated using the classical nonsolvent-induced phase separation method and thermal cross-linking. The effectiveness of membranes was evaluated using dilute Arab extra-light crude oil as a feed and toluene as a dilute. The membranes’ performances were investigated in a dead-end cell at pressures between 2 and 5 bar and at temperatures of between 30 and 65 °C. Since the selectivity of the membrane can be regulated by controlling the cross-linking conditions, a two-stage fractionation process has been proposed. In the first stage, it is possible to separate the larger molecules, such as asphaltene, by using the membrane, which has a thin layer with a looser structure. The permeate obtained in the first stage was utilized as feed in the second stage using the more cross-linked membrane. As a result, more than 90% of the hydrocarbons with carbon numbers below C_10_ were concentrated in the permeate at the second stage. In addition, the filtration experiments with pure crude oil were performed at 90, 120, and 150 °C under 15 bar. Thus, both main approaches to reducing the viscosity of the viscous feed have been successfully applied in this study, namely, dilution with toluene and temperature increase. In both cases, the membranes demonstrated high separation efficiency.

In this work, the separation of used engine oil using a laboratory sample of a porous flat PAN membrane was investigated for the first time. The choice of polymer is justified by the fact that PAN is the second most widely used membrane material for the fabrication of micro- and ultrafiltration membranes. Membranes based on PAN are characterized by high strength, good solvent resistance, thermal and chemical stability, and low cost. Compared to other membrane materials, such as polyvinylidene fluoride, polysulfone, and polyethersulfone, PAN is more hydrophilic and less prone to fouling. Additionally, in our previous studies on the deasphalting of crude oil and oil solutions using PAN membranes, UF membranes based on this material demonstrated high rejection for asphaltenes and good fouling resistance [33,34,35,36,37].

In our recent study, a new approach to the targeted regulation of the porous structure of PAN ultrafiltration membranes by adding acetone to the casting solution has been developed [35]. The membranes were prepared using the NIPS method with water as a non-solvent. The presence of acetone in the casting solutions of PAN in dimethylsulfoxide or N-methyl-2-pyrrolidone was studied with regards to thermodynamical aspects of phase separation of polymeric solutions induced by contact with non-solvent, formation, and performance of porous membranes of ultrafiltration range. It has been shown that varying the casting solution composition results in membranes with different pore sizes from 3.7 to 36 nm. For example, by changing the acetone content at a constant polymer concentration in dimethyl sulfoxide (DMSO), it was possible to reduce the MWCO from 58,000 (without acetone) to 1800 g/mol (with acetone) [35]. Based on the above-described developments from [34], our results on the targeted broad variation of the separation characteristics of PAN membranes may allow for the creation of two or more stage processes for the fractionation of used oil. In this work, PAN ultrafiltration membranes were obtained by the NIPS method from a PAN solution in DMSO without acetone additives as a variant of the membrane for the first stage of purification. It is important to note that the chosen PAN sample was an industrial copolymer poly(acrylonitrile-co-methyl acrylate) [P(AN-co-MA)].

## 2. Materials and Methods

### 2.1. Materials

A commercial copolymer of acrylonitrile (AN) and methyl acrylate (MA) (ratio 92:8), designated as P(AN-co-MA), from JSC “VNIISV” (Tver, Russia), was used. The weight average molecular weight of the P(AN-co-MA), M_w_, was 107 kDa, with a polydispersity index M_w_/M_n_ of 2.31. Samples of used oils were collected from the storage tanks in the maintenance area of passenger cars at “Agat-Center” LLC, Ivanovo, Russia.

### 2.2. Polymer Characterization

Fourier-transform infrared (FTIR) spectroscopy and nuclear magnetic resonance (NMR) spectroscopy were used to confirm the structure of the synthesized polymer. IR-spectra registration was carried out in the reflection mode using an IR microscope HYPERION-2000 conjugated with an IR-Fourier spectrometer IFS 66 v/s Bruker (Ge crystal, scan-50, resolution 2 cm^−1^, range 600–4000 cm^−1^) (Bruker Physik AG, Karlsruhe, Germany). The ^13^C-NMR spectrum of the polymer was recorded on a Bruker AVANCE III HD 400 NMR spectrometer (Rheinstetten, Germany). For this, a polymer solution in dimethyl sulfoxide-d6 with a concentration of 2–4% wt./vol. was prepared. The degree of crystallinity of the polymer was investigated using X-ray diffraction (XRD) analysis. X-ray diffraction spectra were obtained using an X-ray diffractometer Rigaku Rotaflex D/MAX-RC (Rigaku, Tokyo, Japan) at room temperature on Cu-Kα radiation.

### 2.3. Preparation of P(AN-co-MA) Solution

A casting solution containing 13 wt.% P(AN-co-MA) and 87 wt.% solvent was prepared. DMSO (Chemically Pure, produced by Khimmed, Moscow, Russia) was used as the solvent. The solution concentration was chosen based on preliminary experiments and results obtained in the article [46]. The casting solution was prepared in 100 mL glass jars. First, the calculated amount of DMSO was poured into the jar, which was then placed on a magnetic stirrer IKA C-MAG HS 10 (IKA-Werke GmbH & Co. KG, Staufen, Germany) set to a moderate stirring speed. Afterward, the polymer was added to the jar, and the resulting solution was stirred on the magnetic stirrer for 72 h at a speed of 50 rpm at room temperature (20–25 °C). After this period, the solution was treated in a Sapphire TTC ultrasonic bath (PMD) for 30 min to achieve complete homogenization and air bubble removal. The resulting solution was stored in a hermetically sealed jar at room temperature and humidity not exceeding 20% in the dark. The dynamic viscosity of the prepared solution was measured at 20 °C using a Brookfield DV2T-RV viscometer (Ametek Brookfield, Middleboro, MA, USA).

### 2.4. Membrane Fabrication by Phase Inversion Method

**Figure 1:**
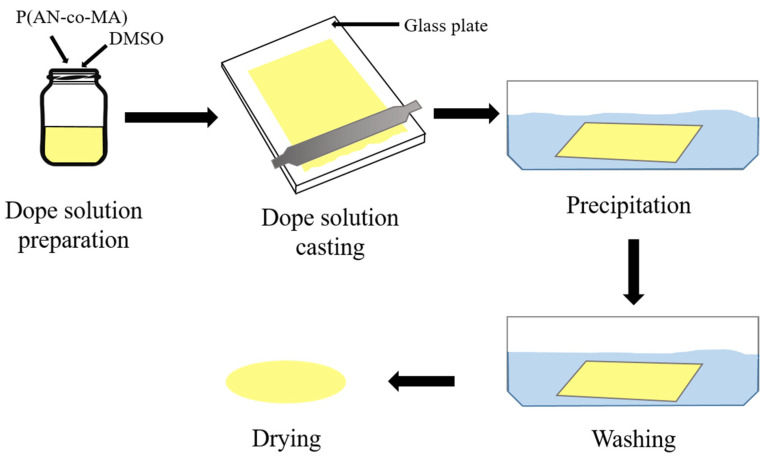
Membrane preparation scheme.

The phase inversion method is the most common technique for the preparation of asymmetric polymeric ultrafiltration membranes. The phase inversion methods include vapor-induced phase separation (VIPS), thermally induced phase separation (TIPS), evaporation-induced phase separation (EIPS), and immersion precipitation or non-solvent-induced phase separation (NIPS). Among these techniques, NIPS is the most commonly employed technique for the preparation of asymmetric polymeric membranes including PAN membranes [47]. In this work, membranes were prepared by the NIPS, with water as a non-solvent (Figure 1). The polymer solution was applied onto acetone-cleaned polished glass using a doctor blade to form a layer with a thickness of 200 µm at a temperature of 20 °C and humidity of 20%. The glass with the applied solution was then quickly immersed in distilled water. After the membrane formation was completed, the samples were transferred to a washing bath filled with distilled water and left for 24 h. The membrane was then placed in ethanol for at least 24 h, followed by an additional 24 h in isobutanol (Chemically Pure, Khimmed, Russia) to prevent pore collapse during drying. After the isobutanol treatment, excess liquid was removed from the membrane using filter paper, and the membrane was left to dry completely at room temperature and 20% humidity in a fume hood, positioned between two sheets of filter paper.

### 2.5. Characterization of P(AN-co-MA) Membranes

The morphology of the membranes was examined using scanning electron microscopy (SEM). SEM was performed on a “Thermo Fisher Phenom XL G2 Desktop SEM” (Massachusetts, MA, USA). Membrane fractures were obtained after pre-soaking the membranes in isopropanol and subsequently breaking them in a liquid nitrogen environment. A thin (5–10 nm) layer of silver was applied to the prepared samples in a vacuum chamber (~0.01 mbar) using a “Cressington 108 auto Sputter Coater” (Redding, CA, UK). The accelerating voltage during the microphotograph capture was 15 kV. The average thickness of the selective layer from the obtained micrographs was determined using the Gwyddion software (ver. 2.53).

The mechanical properties of the P(AN-co-MA) membranes were tested using a TT-1100 tensile machine (Cheminstruments, Fairfield, OH, USA) at room temperature (25 °C), as described elsewhere [48]. The traverse speed was set to 3.8 cm/min. The samples were rectangular pieces of film, approximately 40 mm in length and 10 mm in width, with both ends secured between the clamps. The initial distance between the clamps was 30 mm. The stress calculations were based on the initial cross-sectional area of the sample. Five samples were tested for the calculation of mechanical properties.

The pore size was measured by liquid–liquid porometry using a POROLIQ 1000 ML porometer (Porometer, Nazareth, Belgium). The principle of the device is based on the displacement of a wetting liquid by a non-wetting one. The methodology for determining the pore size of the membrane using liquid–liquid porometry is detailed in the references [36,44]. The main parameter used in this study was the mean flow pore size (MFP), defined as the pore size through which 50% of the flow passes through larger pores and 50% through smaller pores. This measure is higher than the number average pore size, as larger pores contribute more to the overall flow through the membrane. The MFP value is calculated for the pressure at which the permeability is 50% of the maximum value for the given measurement. Simultaneously with the MFP value, the size of the largest pore was determined.

Filtration studies of the membrane were conducted in a dead-end filtration mode. To reduce the effect of concentration polarization during the filtration of used engine oil, continuous stirring at 600 rpm was applied. The membrane sample was placed on porous stainless-steel discs and sealed with rubber O-rings. The active area of the membrane was 7.9 cm^2^. The volume of liquid poured into the cell was 900 mL. The transmembrane pressure was maintained at 3 bar for the filtration of pure solvents and at 15 bar for the separating mixture. Filtration was carried out until a steady-state permeability was reached at a temperature of 25 °C.

The membrane permeability (*P*) was calculated using the following formula [34,35,36,37,49]:

(1) $$P=\frac{m}{\rho \cdot S\cdot \Delta t\cdot \Delta p},$$

where *m* is the mass of permeate (g) passing through the membrane with area *S* (m^2^) during the time Δ*t* (h), ρ is the density of the liquid (g/cm^3^), and Δ*p* is the transmembrane pressure.

Water and toluene permeability and separation characteristics during the filtration of oil solutions were measured. Three independently prepared membrane samples were used for filtration experiments. From each membrane sample, one coupon was cut for the water filtration experiment, and another coupon was used for toluene and UEO filtration. After filtration of toluene (2 h), the UEO solution was filtered (8–11 h). Solutions containing 100 g/L of UEO in toluene were used for filtration. Filtration of the used engine oil solution was carried out until approximately 300 mL of permeate was collected. Initial filtration with toluene was conducted to determine the permeability of the pure solvent. After completing the UEO filtration, the cell was rinsed three times with 50 mL of toluene, and pure toluene was filtered through the fouled membrane for 2 h. The permeability for toluene was determined again to assess fouling during filtration.

The rejection of the membrane (*R*) was determined based on the metal content in the feed (*C_f_*) and in the permeate (*C_p_*) as follows [34,35,36,37,49]:

(2) $$R=\left(1-\frac{C_{p}}{C_{f}}\right)\cdot 100\%$$

To evaluate the fouling parameters of the membrane, four parameters were used: total fouling ratio (*TFR*), reversible fouling ratio (*RFR*), irreversible fouling ratio (*IFR*), and flux recovery ratio (*FRR*) [37,50]. TFR indicates how much the flux of the solution (*J*_S_) was lower than the initial flux of the pure solvent (*J*_1_), reflecting the influence of factors such as gel layer formation, concentration polarization, pore blockage, and others. It should be noted that the viscosity of oil solutions is somewhat higher than that of pure toluene, which undoubtedly affects the transmembrane flux; however, this fact was not considered in the calculations. The *TFR* was calculated as follows [37,50]:

(3) $$TFR=\left(\frac{J_{1}-J_{S}}{J_{1}}\right)\cdot 100\%$$

*RFR* represents the reversible portion of the flux decline, indicating the extent to which the flux of the pure solvent through the membrane fouled as a result of filtration (*J*_2_) exceeds the flux of the solution. This parameter reflects the contribution of reversible factors to the overall fouling, such as concentration polarization and gel layer formation. The *RFR* was calculated as follows [37,50]:

(4) $$RFR=\left(\frac{J_{2}-J_{S}}{J_{1}}\right)\cdot 100\%$$

*IFR* corresponds to the irreversible component of fouling, associated with pore blockage and the formation of a deposit on the membrane surface that does not dissolve upon replacing the oil solution with pure toluene. The *IFR* was calculated as follows [37,50]:

(5) $$IFR=\left(\frac{J_{1}-J_{2}}{J_{1}}\right)\cdot 100\%$$

*FRR* is determined as the ratio of the flux of toluene through the membrane after filtering the oil solution to the flux of toluene through the initial membrane. This parameter, like the *IFR*, indicates the degree of irreversible fouling of the membrane. *FRR* was calculated as follows [37,50]:

(6) $$FRR=\frac{J_{2}}{J_{1}}\cdot 100\%$$

Membrane fouling was further investigated using FTIR spectroscopy in the ATR (Attenuated Total Reflectance) mode. The surface of the membrane was analyzed before and after filtration to conduct a comparative assessment of the obtained spectra. For this, the membrane used in the filtration of the UEO solution was rinsed with toluene and dried to remove any remaining solvent.

### 2.6. Analysis of Oil Composition

For the preliminary assessment of the composition of UEO, permeate, and retentate, gas chromatography coupled with mass spectrometry (GC-MS) was employed. Since UEO was quantitatively diluted with toluene before filtration, no additional sample preparation was required for GC-MS analysis. A Shimadzu GC-2010 Plus gas chromatograph with a mass spectrometric detector (MDGC/GCMS-2010, Shimadzu, Kyoto, Japan) was used. The column was SP-Sil 5 CB (100% polydimethylsiloxane), 30 m in length, 0.32 mm in internal diameter, and 0.25 µm in film thickness. The column thermostat program was as follows: 50 °C (2 min), 4 °C/min, 300 °C (40 min). The mass spectrometer operated in the mass range of 16–600 Da in full ion current mode, with the ionizing electron energy set at 70 eV. The composition of the initial UEO solution, permeate, and retentate was studied according to the methodology described earlier [50]. For this, the total intensities for each fragment ion were extracted from the obtained chromatograms of the samples, and calculations were performed as described below.

The determination of the group composition of complex organic mixtures is based on the use of a homologous series of characteristic fragment ions. The mass spectrum of the mixture represents an additive overlay of the mass spectra of its components. Thus, the total intensity of the peaks of the characteristic ions can be represented as the sum of the intensities of individual types of compounds, taking into account coefficients that consider the mutual overlaps of characteristic sums in the mass spectra of compounds from different groups [50]. Using sensitivity coefficients, which express the dependence of the intensity of characteristic sums on the concentration of the corresponding groups of compounds, the relative content of these groups in the analyzed mixture is determined.

The homologous composition is determined by the intensity of the molecular ion peaks *I_m_* and *I_m_*_−1_ with the introduction of an isotopic correction *K*_1_ and sensitivity coefficients *K*_2_. The concentration of the homolog of the j-th molecular mass is calculated by the formula [50]:

(7) $$P_{j}=\frac{I_{m}-I_{m-1}\cdot K_{1}}{K_{2}},$$

where $P_{j}$ is the concentration of the homolog of the j-th molecular mass, % in the total ion current, *I_m_* is the intensity of the molecular ion, *I_m_*_−1_ is the intensity of the m − 1 ion, *K*_1_ is the isotopic correction, and *K*_2_ is the sensitivity coefficient.

When determining the homologous composition of naphthenic hydrocarbons, the overlap of the peaks of molecular ions of higher and lower homologs, as well as the mass spectra of paraffinic hydrocarbons, is taken into account. The homologous composition of alkylbenzenes is determined by the ion peaks, considering the overlaps of higher members of the homologous series on the molecular ion peaks of benzene and toluene. The ratio of normal and branched paraffinic hydrocarbons is established based on the different intensities of the molecular ion peaks in their mass spectra.

The method for determining normal and branched paraffinic hydrocarbons is based on measuring the total intensity of the characteristic ion peaks (Σ71) and the molecular ion in the mass spectra of normal and branched paraffinic hydrocarbons. To determine the total intensity (Σ71)^n^ attributed to n-paraffinic hydrocarbons in the sample, the corrected values of the molecular ion peak intensities *I_mol_* are multiplied by the corresponding values of Σ71/*I_mol_* and summed. To convert the obtained values (Σ71)^n^ into concentrations, sensitivity coefficients *K_max_* calculated from the spectra of individual hydrocarbons were used [50].

In determining the group composition, the total characteristic intensity of the signals of paraffinic hydrocarbons (Σ71)^n+iso^ was calculated. The quotient of (Σ71)^n^ divided by (Σ71)^n+iso^ determines the share of n-paraffinic hydrocarbons in the analyzed sample.

For further analysis, it was necessary to remove all the toluene from the permeate using distillation. The toluene distillation was carried out on a Hei-VAP Core rotary evaporator (Heidolph Instrument, Schwabach, Germany) with a vacuum pump and a trap cooled with liquid nitrogen. The bath temperature was 90 °C, the flask rotation speed was 14 rpm, and the mixture volume was 600 mL. The distillation process continued until the permeate boiling ceased, and all the toluene was collected in the receiving flask. About 60 mL of filtered oil was obtained at the outlet. Toluene distillation was also performed for the retentate using the same method.

Further, the physicochemical properties of the initial UEO, permeate, and retentate were investigated. The main indicators characterizing possible changes in the composition of the samples are color, optical density, density, dynamic viscosity, and total acid number (TAN). Color determination was carried out according to the ASTM D1500 color scale [51], and density was measured using hydrometers. This choice is explained by the fact that during the operation of engine oil, exposure to oxygen and high temperatures leads to the oxidation and degradation of its components, as well as to the polymerization and condensation of some products. As a result of aging processes, inert substances are formed, primarily alcohols, aldehydes, esters, ketones, asphaltenes, resins, and acidic substances, which cause corrosion and increased metal wear. The optical density was measured using a PE-5400UV spectrophotometer (PromEcoLab, Shanghai, China). Toluene was used as the reference solution. As the wavelength increased, the optical density of the solutions monotonically decreased. For the studied solution concentration of 100 g/L, this optical density was observed at a wavelength of 485 nm. Dynamic viscosity will indicate the degree of removal of undesirable components of used oil, including resin-asphaltene substances, during ultrafiltration [52]. TAN will provide information on the content of acidic substances in the initial mixture, and comparing this indicator in the investigated samples will show whether their removal is possible through the filtration process on P(AN-co-MA) membranes. The total acid number is determined by the potentiometric method according to ASTM D664 or D974 standards [53,54]. Measurements for each solution were carried out at a temperature of 22 °C. ^1^H NMR experiments were conducted for UEO and filtration products. Deuterated chloroform (CDCl_3_) was used as a solvent. Spectra were obtained using a Bruker AVANCE III HD 400 NMR spectrometer (Rheinstetten, Germany). To compare the spectra and qualitatively characterize the changes in composition, solutions of the same concentration were prepared: 20 mg/0.5 mL of solvent.

The metal content in UEO and filtration products (permeate and retentate) was investigated using inductively coupled plasma atomic emission spectrometry (AES) (ICPE-9000, Shimadzu, Kyoto, Japan). The method is based on measuring the emission intensity of atoms of the elements being determined, which occurs when the sample is sprayed into an argon plasma sustained by a radio-frequency electromagnetic field, and it requires preliminary sample preparation. The sensitivity of the AES method allows for the determination of element content from 10^−5^% to 100% with a relative error of ~15%. The sample preparation procedure involved weighing the test samples. The weighed sample was placed in a quartz beaker, treated with 0.1 cm^3^ of concentrated H_2_SO_4_, covered with a watch glass, and dried on a hot plate until a carbonaceous residue formed. The beaker with the samples was placed in a muffle furnace and burned at 500 °C for 5 h. After cooling to room temperature, the resulting ash was mineralized with a mixture of concentrated acids H_2_SO_4_ (1:2) until complete dissolution.

The content of C, H, N, and S in the samples was determined by chromatography after combustion of the sample in a dynamic flash according to the Dumas method. Studies were carried out using a Flash 2000 elemental analyzer (Thermo, Massachusetts, MA, USA). The method involves burning a weighed sample (usually 2–4 mg) at 1700 °C and subsequent chromatographic determination of the resulting oxides: the combustion products pass through a system of redox reactors, are separated on a chromatographic column, and detected by a katharometer.

### 2.7. Dynamic Viscosity Measurements

The dynamic viscosity of the polymer solutions, UEO, permeate, and retentate was measured at 20 °C using a Brookfield DV2T-RV viscometer (Ametek Brookfield, Middleboro, MA, USA).

## 3. Results

### 3.1. Polymer Characterization Results

**Figure 2:**
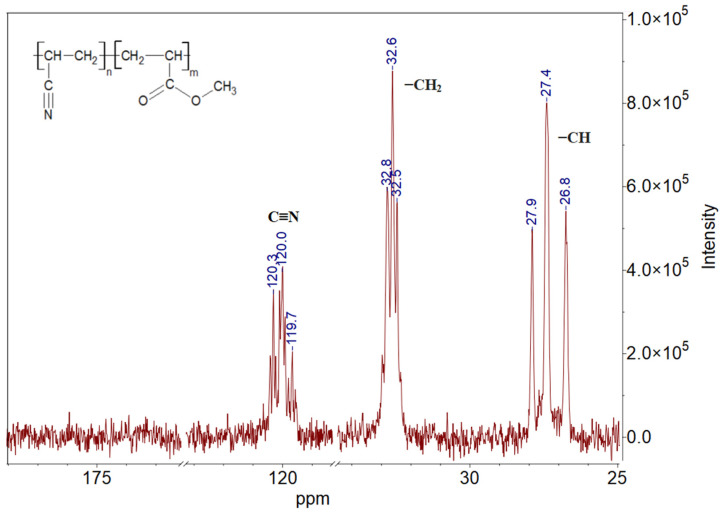
^13^C NMR spectroscopy spectra poly(acrylonitrile-co-methyl acrylate).

The P(AN-co-MA) was characterized using ^13^C NMR spectroscopy (Figure 2). The sample was dissolved in DMSO-d_6_. The broad peak in the range of 26.8–27.9 ppm is attributed to the carbon resonance of the −CH groups of acrylonitrile and methyl acrylate units. The signal associated with the methylene carbon of both comonomers appeared at 32.5–32.8 ppm. Additionally, peaks in the region of 119.7–120.3 ppm were assigned to the carbon in the nitrile group. However, the spectrum lacked the signal associated with the presence of the methoxy group in methyl acrylate, which should be located around ~176 ppm. This could be due to the low concentration of the polymer in the deuterated DMSO solution.

The −CH and C≡N signals are stereospecific and exhibit stereochemical splitting. In the nitrile carbon signal, peaks attributed to iso-, hetero-, and syndiotactic triads manifest from higher to lower magnetic fields, respectively, whereas in the −CH_2_ carbon signal, these peaks manifest from lower to higher magnetic fields, similar to observations made in previous studies [55,56].

**Figure 3:**
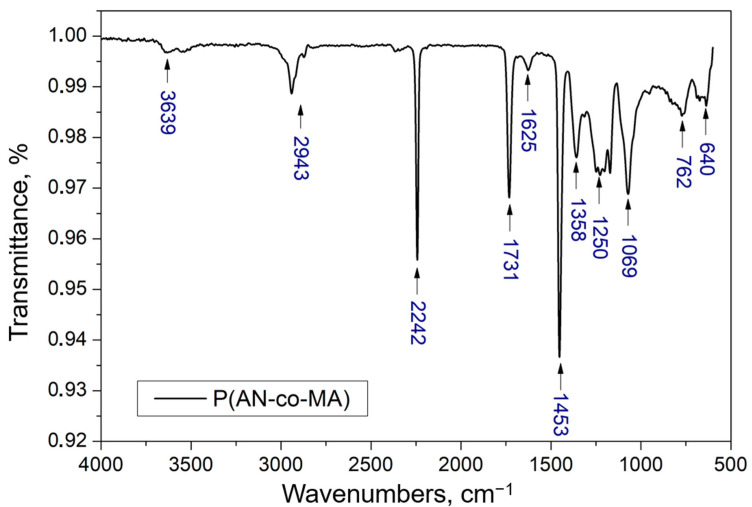
FTIR spectrum of P(AN-co-MA).

The chemical structure of the polymer was further investigated by Fourier-transform infrared (FTIR) spectroscopy (Figure 3). The IR spectrum shows a characteristic of valence vibrations of the nitrile group C≡N at 2242 cm^−1^, which corresponds to the acrylonitrile monomer units. The intense band at 1731 cm^−1^, associated with the carboxyl group C=O, and the broad band at 1250 cm^−1^, related to C−O−C vibrations in esters, confirm the copolymerization of the initial AN and MA monomers. The near absence of the band at 1625 cm^−1^, which characterizes the C=C double bond stretching vibrations, as reported in previous studies [57,58], indicates that the copolymerization process was achieved through the breaking of double bonds in both AN and MA monomers [37].

Bending vibrations of the methylene group −CH_2_ are observed in the regions of 1358 and 1453 cm^−1^, while the band around 1069 cm^−1^ is attributed to mixed vibrations of the −CH_2_ group [59,60]. The band at 762 cm^−1^ can be ascribed to mixed vibrations of the C−H bond [55,60,61]. The broad band in the range of 3100–2700 cm^−1^ with a maximum at 2935 cm^−1^ corresponds to the stretching of C−H in CH, CH_2_, and CH_3_ [55]. A band is also observed in the 3700–3600 cm^−1^ range, corresponding to the O-H group, indicating the presence of residual water in the polymer.

**Figure 4:**
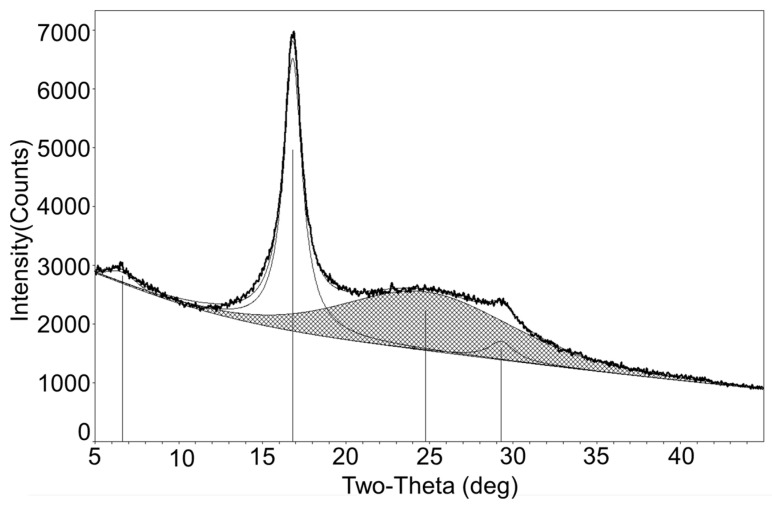
XRD spectrum of polymer: “crystalline” peaks—transparent, “amorphous”—shaded.

The XRD analysis of the P(AN-co-MA) shows that the most intense peak is observed at 2θ = 16.79° (Figure 4), which corresponds to the (100) plane of the hexagonal lattice of the polymer. The second peak, corresponding to the (101) plane, is observed at 2θ = 29.43° [62]. The degree of crystallinity was calculated as the ratio of the areas of the “crystalline” peaks to the total peak area, which was 45%.

### 3.2. Preparation and Characterization of Membranes

**Figure 5:**
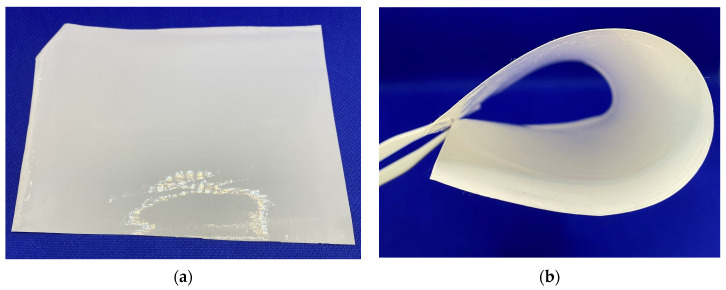
Asymmetric ultrafiltration P(AN-co-MA) membrane images. (**a**) the original membrane, (**b**) demonstration of membrane flexibility.

Dynamic viscosity is a crucial parameter that significantly determines membrane properties. The viscosity of the casting solution with a polymer content of 13% was 89.53 Pa·s. Flat membranes with a thickness of 90 μm were obtained from this solution (Figure 5).

**Figure 6:**
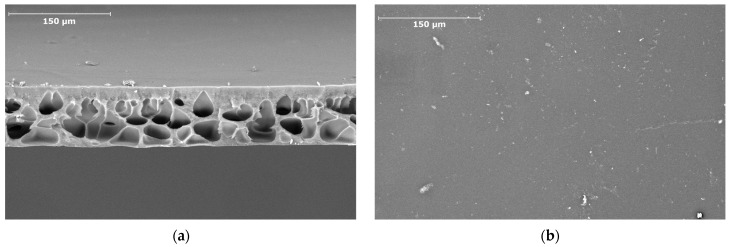
SEM images of the cross-section (**a**) and the surface (**b**) of the P(AN-co-MA) membrane.

The analysis of the SEM image of the membrane cross-section showed that the membrane had an asymmetric structure with cellular macrovoids and a thin surface skin layer (Figure 6a). The thickness of the P(AN-co-MA) skin layer was approximately 6 μm. The SEM image of the surface showed no significant defects, and the surface pore size of the skin layer was substantially below the resolution limit of the used method (Figure 6b). On the other hand, according to liquid–liquid porometry data, the average pore size of the membrane was 23.1 nm, with the largest pore size being 73.4 nm and the smallest one 16.2 nm. The porometry results of transport pores in the membrane also confirmed the absence of significant defects in the membranes.

The mechanical strength and elastic properties of the membrane were analyzed (Table 1). The membranes show satisfactory mechanical properties: tensile strength of 7.2 MPa, Young’s modulus of 152 MPa, and elongation at break of 8%. This ensured the necessary strength and flexibility of the membrane during its operation (Figure 5).

### 3.3. Ultrafiltration of Used Engine Oil

During the water filtration process, a decrease in membrane permeability was observed, followed by a transition to a steady-state flow regime characterized by constant permeability over time. The steady permeance value was achieved after 1 h of filtration, with a permeance of 54.15 L/m^2^·h·bar, and remained unchanged for one hour thereafter (Table 2). In the case of toluene, the steady-state filtration regime was established only after 2 h of filtration, and the transition from water to nonpolar solvent was marked by a 69.6% reduction in permeability.

**Figure 7:**
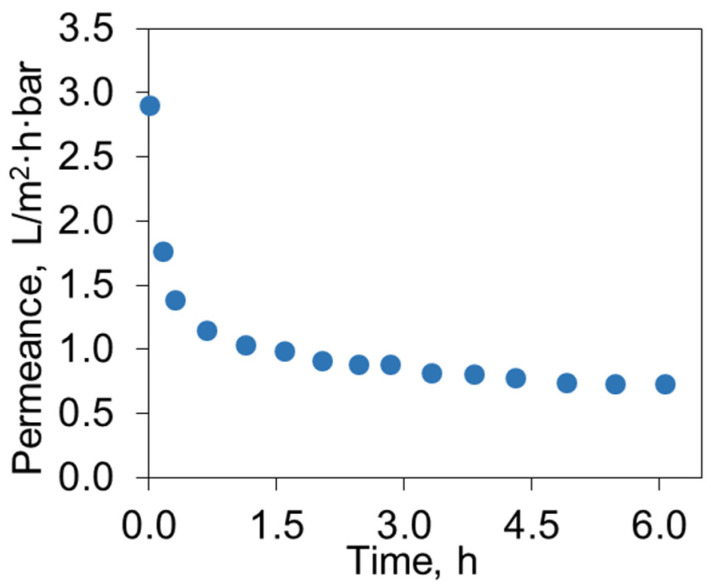
Time dependence of the UEO solution permeance through the P(AN-co-MA) membrane.

Filtration of a 100 g/L used engine oil solution in toluene was carried out for 11–13 h to obtain the required volume of permeate (Figure 7). Upon transitioning from toluene to oil solutions, a sharp decline in permeability was observed within the first 70 min, followed by a gradual decrease in permeance because of fouling. The permeance of the UEO solution in toluene (0.75 L/(m^2^·h·bar) at 25 °C) was higher than the permeance of waste lubricant oil through a PAN ultrafiltration membrane at 40 °C (~0.1 L/(m^2^·h·bar)) without dilution [39].

**Figure 8:**
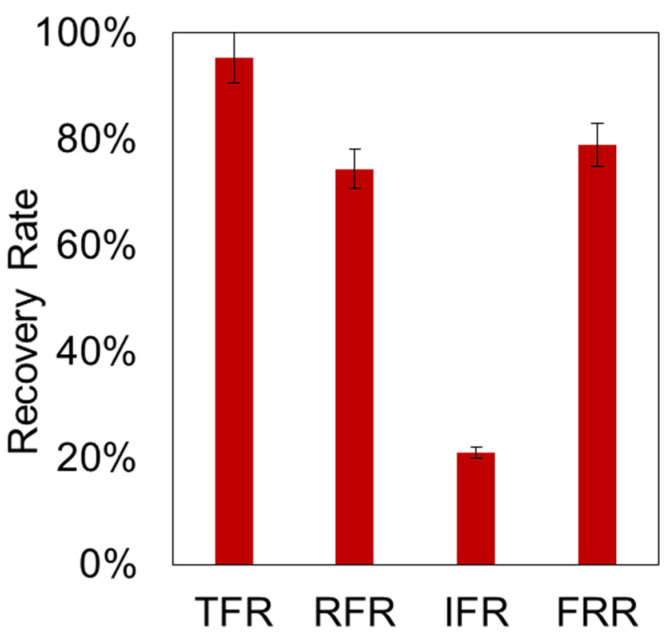
The recovery rate of the membrane fouled during filtration of UEO solution in toluene (100 g/L).

Fouling of membranes and the development of methods for their regeneration are the most important criteria for the successful application of an ultrafiltration membrane in real processes [63]. Therefore, considering resistance as an important parameter of the developed P(AN-co-MA) membranes, the values of TFR, RFR, IFR, and FRR were measured to evaluate their antifouling property as a crucial parameter (Figure 8).

The total fouling ratio of the membrane is 95%, which can be attributed to concentration polarization. The permeability of the membrane during the filtration of used engine oil solutions was 20 times lower than the permeability of pure toluene. However, rinsing the membrane with toluene showed that most of the fouling was reversible. This may indicate that the main contribution to fouling is the effect of clogging pores on the membrane surface. This is evidenced by the IFR and RFR values, which are 21% and 74%, respectively. At the same time, the flux recovery ratio (FRR) is 79%, indicating a high resistance of the membrane to fouling.

### 3.4. FTIR Spectroscopy

**Figure 9:**
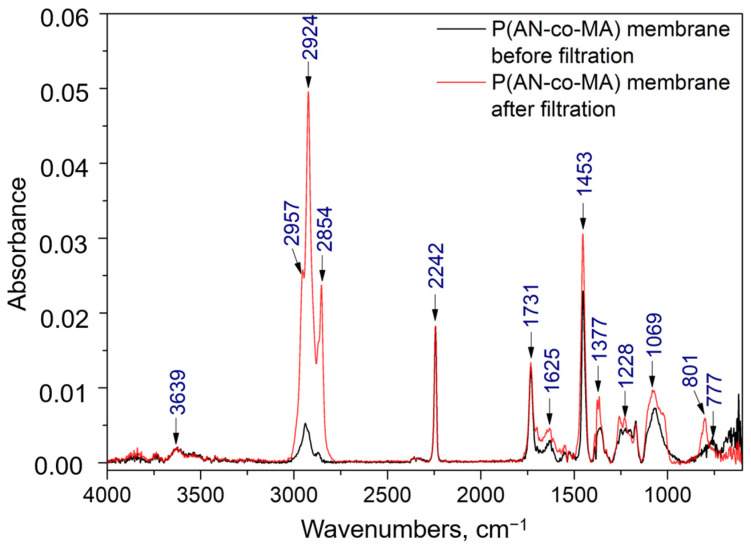
FTIR spectrum of the P(AN-co-MA) membrane surface before and after filtering.

To confirm membrane fouling, we studied the membrane surface before and after filtration of the UEO solution in toluene using FTIR spectroscopy (Figure 9). The FTIR spectra of the membranes before and after filtration contain all the characteristic bands of the P(AN-co-MA) polymer: stretching vibrations of C-H are observed in the 2950–2850 cm^−1^ range, the nitrile group (C≡N) at 2242 cm^−1^, C-H deformations at ~1453 and 1377 cm^−1^, mixed C-H and wagging CH_2_ at ~1069 cm^−1^, weak absorption bands at 1228 and 777 cm^−1^, stretching vibrations of C=O at 1731 cm^−1^, and mixed C-O stretching and deformation vibrations from C=O and C-O in the 1250–1000 cm^−1^ range from methyl acrylate [64]. The band in the 3700–3600 cm^−1^ range characterizes the O-H group, indicating residual water or alcohol on the membrane surface, which was also observed in the FTIR spectrum of the polymer.

For comparison, the spectra were normalized to the C≡N group, as this group did not undergo changes during filtration. The IR spectra of the membrane before and after filtration show some changes related to membrane fouling by aliphatic and aromatic compounds. The most intense bands in the regions of 2957 cm^−1^, 2924 cm^−1^, and 2854 cm^−1^ [65] correspond to the symmetric stretching of the C–H group in alkanes, and there is an increase in the intensity of the 1453 cm^−1^ band. Additionally, C-H deformation vibrations at 801 cm^−1^ from disubstituted aromatic compounds are observed.

### 3.5. Some Physicochemical Properties of Used Engine Oil, Permeate, and Retentate

**Figure 10:**
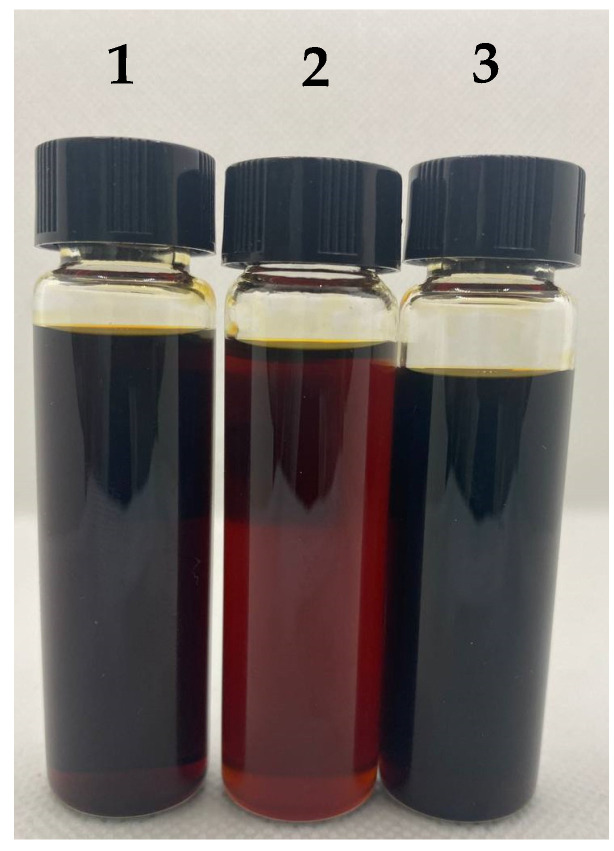
Photographs of (1) feed (UEO as received), (2) permeate, and (3) retentate after filtrations of UEO solutions in toluene (100 g/L): permeate and retentate after removal of toluene by distillation.

In Figure 10, the photographs of UEO, permeate, and retentate after filtrations of UEO solutions in toluene (100 g/L). It can be seen that the permeate is transparent, and its color is brown, corresponding to 5.5 on the color scale, while UEO and retentate are opaque and dark, corresponding to 6.5 on the color scale. This indicates that most contaminants can be effectively removed during filtration through the P(AN-co-MA) membrane.

To further evaluate the effectiveness of ultrafiltration separation using P(AN-co-MA) membranes, the physicochemical properties of UEO, permeate, and retentate were measured. The optical density was determined in diluted samples, and for measuring density, color, dynamic viscosity, and acid number, toluene was removed from the permeate and retentate by distillation. As can be seen from Table 3, a significant improvement in the quality of the permeate compared to UEO and retentate was observed. During the filtration process, the dynamic viscosity of the permeate decreased, and the retentate increased in comparison with the UEO. Moreover, the level of reduction of this parameter for permeate (from 59 to 49 MPa·s) is significantly higher than its increase for retentate (from 59 to 61 MPa·s). Similar, and even much more significant, changes are observed for the optical density parameter, which indirectly confirms the rejection of various solid particles and polymerization products by the membrane, which are formed during the degradation of engine oil. So, this value is 0.690 and 1.024 for permeate and UEO, respectively.

Some of the oil molecules will oxidize to form corrosive organic acids at higher temperatures inside the engine; therefore, the acidity of UEO is the major property in determining the quality of oils [1,65]. As can be seen from Table 3, the total acid number decreases in permeate after ultrafiltration from 0.43 to 0.33 mg KOH/g. Acids form in the initial stage of hydrocarbon oxidation and cause resinous component formation, which increases oil viscosity and forms various deposits on heated surfaces, leading to increased engine wear. The formation of weak organic acids during oil oxidation contributes to the corrosion of parts and the degradation of rubber seals, while resinous components form various deposits that increase engine wear. The oxidation of oil is accelerated by metal particles and inorganic contaminants, which accumulate in the oil mainly as a result of engine part wear. At the same time, if the total acid number value of the used engine oil corresponds to the standards of fresh engine oil, indicating a low degree of aging of the studied UEO [66].

### 3.6. Elemental Analysis of Used Oil, Permeate, and Retentate

**Figure 11:**
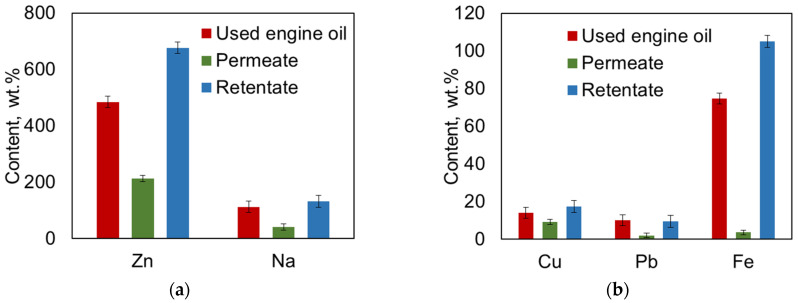
Metal content in the UEO, permeate, and retentate. (**a**) Zn and Na, (**b**) Cu, Pb and Fe.

Elemental analysis for C, H, N, and S showed that the content of these nonmetals in UEO before and after filtration remained unchanged: carbon content was 84.64 wt.%, hydrogen content was 14.15 wt.%, and sulfur content was 0.16 wt.%. Nitrogen content was below the detection limit of the instrument. Elemental analysis of metals indicated a decrease in the concentrations of Cu, Na, Pb, Fe, and Zn in the permeate during the membrane separation process (Figure 11a,b).

The results demonstrated that the P(AN-co-MA) membranes are highly efficient in terms of metal removal; in particular, Fe rejection was at a high level (>96.3%). It is also important to emphasize that since the content of nonmetals in UEO before and after filtration remained unchanged, the P(AN-co-MA) membrane purifies the used oil from unwanted components while maintaining the carbon and hydrogen content in the oil. This indicates that the membrane allows most hydrocarbons, which constitute the base of engine oil, to pass through.

### 3.7. Structural Group Analysis by Mass Spectrometry

**Figure 12:**
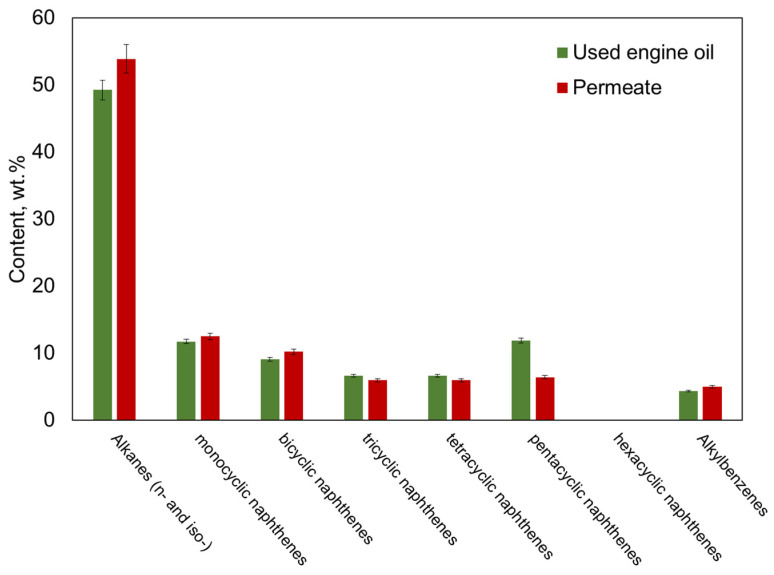
Group hydrocarbon composition of UEO and permeate.

Structural group analysis of UEO and filtration products was conducted using the methodology described in Section 3 [50]. According to the hydrocarbon group composition (Figure 12), the used engine oil is highly aromatic, containing 4.96 wt.% alkylbenzenes and a low saturated fraction content of 49.21 wt.%. The naphthenic fraction accounts for 45.83 wt.%, with a distribution showing a predominance of mono-, bi-, and pentacyclic naphthenes, while hexacyclic naphthenes are completely absent. After filtration, the permeate shows an increase in the content of paraffinic and mono-, bicyclic naphthenic hydrocarbons and aromatic hydrocarbons, with a decrease in the fraction of naphthenes with 3–5 rings in the molecule. The significant difference in the chemical compositions of the UEO and the permeate is observed in the content of pentacyclic naphthenes, indicating improved oil quality after the filtration process.

### 3.8. GS-MS Analysis of UEO, Permeate, and Retentate

**Figure 13:**
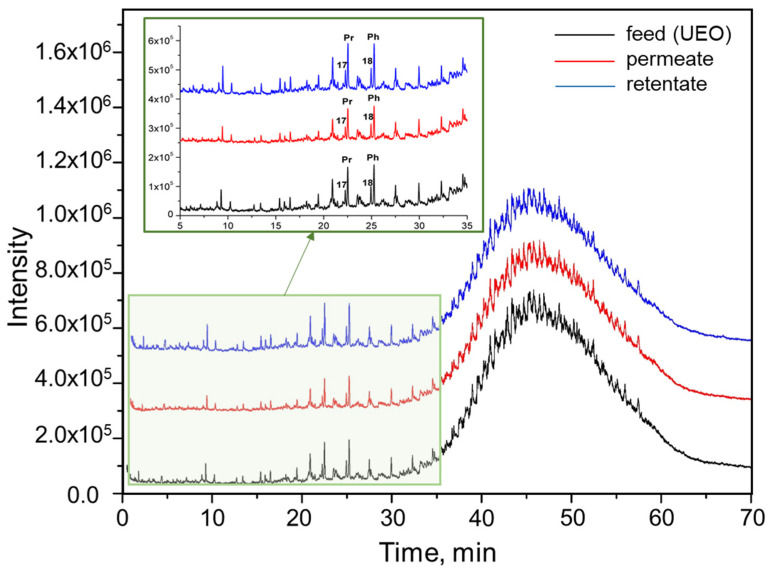
Chromatograms of used engine oil, permeate, and retentate obtained by the fingerprint method during filtration through a P(AN-co-MA) membrane. Conditions: 50 °C (2 min), 4 °C/min, 300 °C (40 min); carrier gas—helium, column SP-Sil 5 CB; inlet column pressure: 312.8 kPa.

The composition of the samples of used engine oil, permeate, and retentate were also analyzed using GC-MS. Data comparison was conducted using the fingerprint method (Figure 13). Differences in the chromatograms of the samples were observed in the 5–35 min range, corresponding to the elution range of light hydrocarbons, cyclic paraffins, and aromatics [65,67]. The samples contained linear alkanes, and peaks of n-heptadecane, n-octadecane, and isoprenoid alkanes, pristane, and phytane were clearly observed.

Table 4 presents the peak area ratios of isoprenoids (pristane and phytane) to the peak areas of linear alkanes chromatographed with isoprenoids and the peak area ratios of pristane to phytane. As seen in the table, the peak area ratios for pristane/heptadecane and phytane/octadecane do not change within the margin of error for the retentate and permeate. Thus, we can conclude that in the process of filtration of UEO solutions in toluene, there is no depletion of permeate by branched hydrocarbons.

It is important to note that the removal of light hydrocarbons from UEO is planned at the next stage of the purification process by using an ultrafiltration PAN membrane with a smaller pore size and narrower pore size distribution, developed in our recent publication [35].

### 3.9. NMR Analysis of UEO and Filtration Products

**Figure 14:**
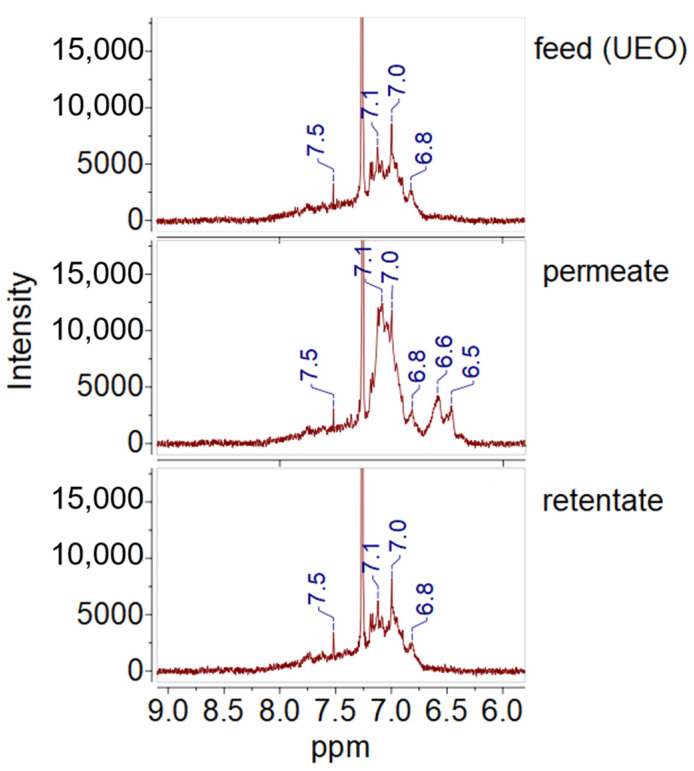
^1^H NMR spectroscopy spectra of oils (9.0−6.0 ppm): feed, permeate, and retentate.

**Figure 15:**
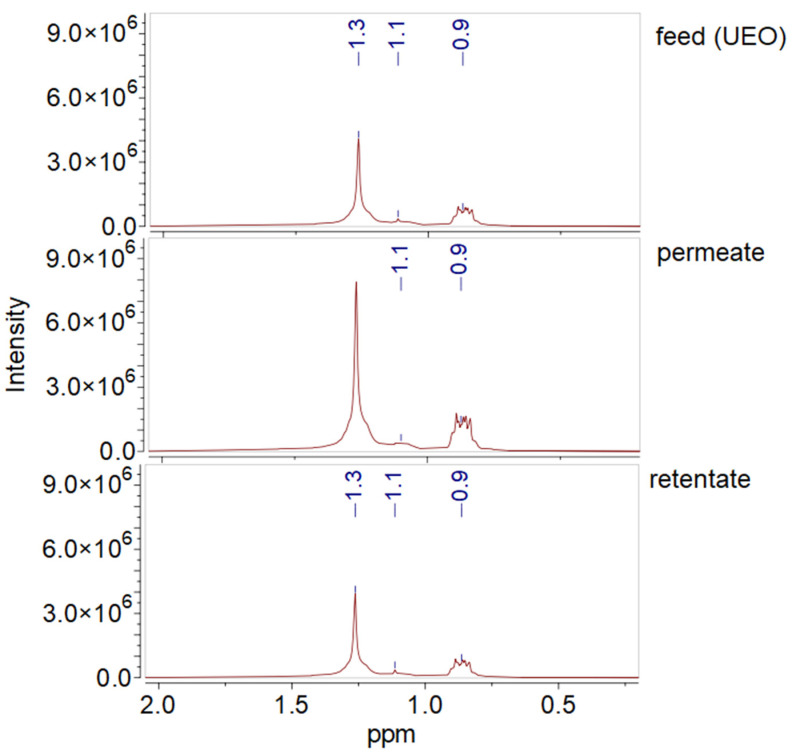
^1^H NMR spectroscopy spectra of oils (2.0−0.0 ppm): feed, permeate, and retentate.

^1^H-NMR analysis of UEO, permeate, and retentate (Figure 14 and Figure 15) provided more detailed information on changes in carbon composition. The spectra of the analyzed samples showed a low-intensity signal at δ ≈ 7.5 ppm, corresponding to phenols. The intensity of this chemical shift did not change in permeate, indicating that phenolic-type antioxidants were not entirely consumed during engine oil use and were not retained by the P(AN-co-MA) membrane. All three spectra showed broad peaks with a chemical shift of 6.8–7.1 ppm [67], corresponding to the presence of aromatic hydrocarbons, which is consistent with the GC-MS and the structural analysis data. The intensity of this peak is higher in the permeate than in the UEO and retentate, indicating an increase in the content of aromatic compounds during filtration. These results, indicating the enrichment of permeate with aromatics, are in good agreement with the data of the structural group analysis (Section 3.6). Notably, aromatic hydrocarbons are added to engine oils as various additives, such as corrosion and oxidation inhibitors or multifunctional antioxidants, anti-wear agents, and to improve the viscosity index of lubricants [67]. The spectra show no signals in the chemical shift range of 8.0–9.5 ppm, indicating the absence of polycyclic aromatic hydrocarbons [68,69]. The aromatic compounds, the content of which increases in the permeate, are primarily represented by alkylbenzenes, which are desirable components in engine oils.

Figure 15 presents the spectra of the analyzed samples of UEO, permeate, and retentate in the 2.0–0.0 ppm range. Methyl and methylene proton peaks were observed in the 0.95–0.8 ppm and 1.3 ppm ranges, respectively [67]. A low-intensity signal with a chemical shift of 1.1 ppm corresponds to the t-butyl groups of hydrocarbons. The analyzed spectra of the samples did not show changes in the intensity of the t-butyl group peak. A significant difference in the concentration of paraffinic hydrocarbons between the feed (UEO) and permeate samples was observed, with their higher concentration in the permeate, which was also revealed by the structural group analysis.

NMR results show that when UEO solutions in toluene are filtered through a P(AN-co-MA) membrane, the permeate is enriched with both long-chain saturated hydrocarbons and alkylbenzenes.

## 4. Conclusions

In this study, the treatment of used engine oil diluted using toluene with the ultrafiltration process was investigated, and special attention was given to the evaluation of membrane fouling and recovery of membrane performance after its cleaning with toluene washing. The commercial copolymer of poly(acrylonitrile-co-methyl acrylate), referred to as P(AN-co-MA), with M_w_ of 107 kDa, and M_w_/M_n_ of 2.31 was selected as a membrane material due to its relatively low cost, and good chemical stability in aliphatic and aromatic hydrocarbons such as engine oil, and toluene. The ultrafiltration membrane with asymmetric porous structure was fabricated by a non-solvent-induced phase separation method when the thin film of 13 wt.% of P(AN-co-MA) solution in DMSO deposited on the glass was precipitated in a water bath. The resulting membrane was characterized by a mean pore size of 23.1 nm, a water permeability of 54.15 ± 0.34 L/m^2^·h·bar, and a toluene permeability of 16.48 ± 0.15 L/m^2^·h·bar.

The filtration of UEO solution in toluene (100 g/L) revealed a sharp decline in membrane performance, followed by a gradual decrease of permeance because of fouling. The overall membrane fouling was 95%. It was shown that the membrane washing with toluene allowed the restororation of up to 79% of the initial permeability, indicating good membrane resistance toward fouling. The IR spectra of the membrane before and after filtration plus rinsing in toluene showed some changes related to irreversible membrane fouling by aliphatic and aromatic compounds. The composition analysis of UEO before and after filtration showed a reduction of metal content in permeate, while the carbon and hydrogen content remained unchanged. According to group hydrocarbon analysis and NMR spectroscopy, the ultrafiltration process of the used engine oil results in a decrease in the proportion of polycyclic naphthenic hydrocarbons and an increase in the proportion of saturated hydrocarbons and alkylbenzenes, which leads to an improvement in the oil quality. The study of the physicochemical properties of the UEO and permeate showed a decrease in optical density from 1.024 to 0.69, dynamic viscosity from 59 to 49 mPa·s, and acid number from 0.43 to 0.33 mg KOH/g. These data indicate the rejection of various solid particles, polymerization products, and oxidation products during filtration. Thus, it was demonstrated that PAN membranes effectively remove the main impurities from used engine oil, primarily polymerization products, and metals, with the possibility of subsequent additional purification and further use of purified lube oil.

## Figures and Tables

**Table 1:** Mechanical properties of the P(AN-co-MA) membrane.

Mechanical Properties	Values
Tensile strength, MPa	7.2 ± 0.6
Young’s modulus, MPa	152 ± 24
Elongation at break, %	8 ± 2

**Table 2:** Characteristics of the P(AN-co-MA) membrane in terms of permeability of various solutions.

Filtration Characteristics	Values
Water permeance, L/m^2^·h·bar	54.15 ± 0.34
Toluene permeance, L/m^2^·h·bar	16.48 ± 0.15
UEO solution permeance (100 g/L in touene), L/m^2^·h·bar	0.75 ± 0.03

**Table 3:** Physicochemical parameters of used oil, permeate, and retentate.

Samples	Optical Density	Density, g/sm^3^	Dynamic Viscosity, mPa·s	The Acid Number, mg KOH/g
Used oil	1.024 ± 0.008	0.856 ± 0.001	59 ± 2	0.43 ± 0.03
Permeate	0.690 ± 0.050	0.854 ± 0.001	49 ± 1	0.33 ± 0.02
Retentate	1.199 ± 0.003	0.858 ± 0.002	61 ± 2	0.45 ± 0.02

**Table 4:** Ratios of peak areas of isoprenoids and linear alkanes in used engine oil, permeate, and retentate.

Samples	S(Pr)/S(C_17_H_36_)	S(Ph)/S(C_18_H_38_)	S(Pr)/S(Ph)
Used oil	2.80 ± 0.10	2.7 ± 0.1	0.94 ± 0.01
Permeate	2.80 ± 0.20	2.8 ± 0.3	0.89 ± 0.04
Retentate	2.79 ± 0.03	2.7 ± 0.3	0.90 ± 0.10

## Data Availability

Data are contained within the article.

## Abbreviations

AES	atomic emission spectrometry;
AN	acrylonitrile;
C_f_	metal content in the cell;
C_p_	metal content in the permeate;
DMSO	dimethyl sulfoxide;
FRR	flux recovery ratio;
FTIR	Fourier-transform infrared;
GC-MS	gas chromatography–mass spectrometry;
GE	grapheme;
GmbH	Gesellschaft mit beschränkter Haftung;
HFCMs	hollow fiber composite membranes;
I	intensity of the molecular ion peak;
IFR	irreversible fouling ratio;
J	liquid flux;
K	coefficient;
KAUST	King Abdullah University of Science and Technology;
m	mass;
MA	methyl acrylate;
MD	mass spectrometric detector;
M_n_	number average molecular weight;
M_w_	weight average molecular weight;
MFP	mean flow pore size;
MWCO	molecular weight cut-off;
NIPS	non-solvent induced phase separation;
NMR	nuclear magnetic resonance;
P	permeability;
∆p	trans-membrane pressure;
PAN	polyacrylonitrile;
P(AN-co-MA)	poly(acrylonitrile-co-methyl acrylate);
PES	polyethersulphone;
PGF	particle glass fibers;
PI	polyimide;
PP	polypropylene;
PTFE	polytetrafluoroethylene;
PVDF	polyvinylidenefluoride;
R	rejection;
RFR	reversible fouling ratio;
SEM	scanning electron microscopy;
t	filtration time;
TAN	total acid number;
TFR	total fouling ratio;
ρ	density;
UEO	used engine oil;
V-SEP	Vibratory Shear Enhanced Process;
XRD	X-ray diffraction.
